# Supplementary material for: Microbial communities mediating algal detritus turnover under anaerobic conditions
Source: PeerJ. 2017 Jan 10;5:e2803. doi: 10.7717/peerj.2803 (PMC5228501; doi:10.7717/peerj.2803)

Supplementary material for:

**Microbial communities mediating algal detritus turnover under anaerobic conditions**

Jessica M. Morrison*^1^, Chelsea L. Murphy*^1^, Kristina Baker^1^, Richard M. Zamor^2^, Steve Nikolai^2^, Shawn Wilder^3^, Mostafa S. Elshahed^1^, Noha H. Youssef^1^

^1^Department of Microbiology and Molecular Genetics, Oklahoma State University, Stillwater, OK, USA

^2^Grand River Dam Authority (GRDA), Vinita, OK, USA

^3^Department of Integrative Biology, Oklahoma State University, Stillwater, OK, USA

Running Title: Degradation of algal biomass by anaerobes

Table S1. Number of high-quality sequences, and observed OTU_0.03_ and OTU_0.1_ obtained in this study. Datasets are grouped by the inoculum source then by algae type used then by week of enrichment. Species richness estimates (Chao and ACE) and Good’s coverage are also shown for each dataset.

| Enrichment Source | Algae type | Week of enrichment | # of sequences | 0.03 | | | | 0.1 | | | |
| --- | --- | --- | --- | --- | --- | --- | --- | --- | --- | --- | --- |
|  |  |  |  | Coverage | OTUs | Chao | ACE | Coverage | OTUs | Chao | ACE |
| ZDT | None | 0 | 17,310 | 0.898 | 3337 | 6449 | 8674 | 0.982 | 876 | 1346 | 1525 |
|  | Chara | 7 | 54,440 | 0.99 | 1167 | 2155 | 3031 | 0.998 | 427 | 589 | 566 |
|  |  | 10 | 29,906 | 0.987 | 888 | 1531 | 2030 | 0.996 | 365 | 508 | 478 |
|  |  | 16 | 39,294 | 0.986 | 1114 | 2323 | 3079 | 0.996 | 433 | 625 | 739 |
|  | Chlorella | 7 | 16,398 | 0.987 | 506 | 892 | 1091 | 0.994 | 259 | 381 | 464 |
|  |  | 10 | 39,740 | 0.989 | 873 | 1691 | 2305 | 0.997 | 373 | 528 | 612 |
|  |  | 16 | 50,640 | 0.988 | 1282 | 2560 | 3474 | 0.997 | 454 | 631 | 704 |
|  | Kelp | 7 | 15,701 | 0.962 | 943 | 2045 | 4004 | 0.988 | 407 | 672 | 904 |
|  |  | 10 | 16,459 | 0.957 | 1133 | 2442 | 4358 | 0.989 | 433 | 697 | 874 |
|  |  | 16 | 18,341 | 0.969 | 846 | 2171 | 3810 | 0.991 | 357 | 536 | 735 |
| WWT | None | 0 | 16,348 | 0.977 | 792 | 1423 | 1770 | 0.995 | 215 | 358 | 479 |
|  | Chara | 7 | 17,216 | 0.981 | 735 | 1240 | 1588 | 0.995 | 315 | 424 | 412 |
|  |  | 10 | 82,226 | 0.991 | 1606 | 2945 | 3799 | 0.998 | 497 | 704 | 759 |
|  |  | 16 | 39,150 | 0.987 | 1187 | 1997 | 2650 | 0.997 | 416 | 586 | 669 |
|  | Chlorella | 7 | 9,827 | 0.971 | 645 | 1076 | 1372 | 0.990 | 293 | 425 | 468 |
|  |  | 10 | 28,353 | 0.987 | 928 | 1468 | 1721 | 0.996 | 363 | 547 | 549 |
|  |  | 16 | 52,042 | 0.991 | 1090 | 1887 | 2214 | 0.998 | 407 | 528 | 515 |
|  | Kelp | 7 | 50,137 | 0.99 | 645 | 1959 | 2780 | 0.998 | 313 | 474 | 612 |
|  |  | 10 | 42,544 | 0.989 | 854 | 1820 | 2718 | 0.997 | 267 | 429 | 585 |
|  |  | 16 | 52,653 | 0.994 | 620 | 1312 | 1837 | 0.998 | 206 | 359 | 458 |
| GL | None | 0 | 12,320 | 0.974 | 527 | 1120 | 1729 | 0.991 | 249 | 397 | 512 |
|  | Chara | 4 | 33,765 | 0.996 | 263 | 657 | 1114 | 0.998 | 118 | 228 | 315 |
|  |  | 8 | 17,584 | 0.993 | 244 | 563 | 776 | 0.997 | 114 | 261 | 346 |
|  |  | 13 | 6,945 | 0.989 | 154 | 379 | 396 | 0.997 | 71 | 106 | 98 |
|  | Chlorella | 4 | 14,464 | 0.993 | 209 | 365 | 506 | 0.996 | 119 | 214 | 316 |
|  |  | 8 | 23,447 | 0.995 | 244 | 458 | 553 | 0.998 | 125 | 208 | 241 |
|  |  | 13 | 28,407 | 0.995 | 307 | 518 | 812 | 0.998 | 153 | 213 | 222 |
|  | Kelp | 4 | 132 | 0.788 | 39 | 228 | 329 | 0.848 | 33 | 81 | 140 |
|  |  | 8 | 3,971 | 0.968 | 366 | 459 | 488 | 0.987 | 202 | 230 | 241 |
|  |  | 13 | 18,027 | 0.983 | 655 | 1127 | 1375 | 0.994 | 306 | 463 | 526 |

Table S2. Detailed bacterial and archaeal community composition in the 9 microcosms studied as compared to the source inoculum (T_0_). Datasets are grouped by the inoculum source then by algae type used then by week of enrichment. Values are percentages of total sequences affiliated with Bacteria or Archaea. The number of sequences affiliated with Archaea in all GL enrichments and inmost of the WWT enrichments were either completely absent or were <50 sequences total and so archaeal community composition is not reported in these datasets.

| Inoculum source | Phyla | T_0_ | Chara | | | Chlorella | | | Kelp | | |
| --- | --- | --- | --- | --- | --- | --- | --- | --- | --- | --- | --- |
|  |  |  | T_7_ | T_10_ | T_16_ | T_7_ | T_10_ | T_16_ | T_7_ | T_10_ | T_16_ |
| ZDT | **Archaeal phyla/classes** | | | | | | | | | | |
|  | Methanobacteria | 0.478 | 0 | 0 | 0 | 0 | 0 | 0 | 0 | 0 | 0 |
|  | Methanococci | 0.957 | 0 | 0 | 0 | 79.660 | 0 | 3.090 | 1.720 | 0.850 | 0 |
|  | Methanomicrobia | 7.177 | 78.470 | 69.880 | 31.940 | 13.560 | 61.700 | 42.270 | 12.070 | 21.370 | 16.360 |
|  | Thermoplasmata | 8.612 | 9.030 | 8.430 | 18.750 | 3.390 | 2.130 | 6.190 | 8.620 | 8.550 | 7.270 |
|  | Other Euryarchaeota | 10.048 | 0 | 0 | 0.690 | 0 | 4.260 | 11.340 | 3.450 | 3.420 | 0 |
|  | Bathyarchaeota | 12.440 | 8.330 | 13.250 | 40.280 | 0 | 8.510 | 21.650 | 3.450 | 0.850 | 3.640 |
|  | Thaumarchaeota | 54.060 | 4.170 | 7.230 | 8.330 | 3.390 | 21.280 | 14.430 | 67.240 | 63.250 | 72.730 |
|  | Unclassified Archaea | 6.220 | 0 | 0 | 0 | 0 | 2.130 | 1.040 | 3.450 | 1.710 | 0 |
|  | **Bacterial phyla/classes** | | | | | | | | | | |
|  | Acidobacteria | 1.524 | 0.037 | 0.073 | 0.118 | 0.019 | 0.031 | 0.039 | 0.105 | 0.311 | 0.052 |
|  | Actinobacteria | 10.795 | 0.133 | 0.209 | 0.145 | 0.224 | 0.597 | 0.661 | 2.445 | 3.872 | 3.741 |
|  | BD1-5 | 0.006 | 0.023 | 0.007 | 0 | 0 | 0 | 0 | 0 | 0 | 0 |
|  | Bacteroidetes | 17.321 | 30.565 | 29.523 | 39.186 | 12.659 | 10.133 | 17.345 | 5.822 | 1.768 | 1.094 |
|  | Caldiserica | 0 | 0 | 0 | 0 | 0 | 0.005 | 0.047 | 0 | 0 | 0 |
|  | Candidate_division_BRC1 | 0 | 0.023 | 0.017 | 0.018 | 0.025 | 0.010 | 0.008 | 0 | 0 | 0 |
|  | Candidate_division_JS1 | 0.095 | 0.002 | 0 | 0 | 0 | 0.005 | 0.008 | 0.013 | 0.006 | 0 |
|  | Candidate_division_OD1 | 0.006 | 0.002 | 0 | 0 | 0 | 0 | 0 | 0.007 | 0 | 0 |
|  | Candidate_division_OP11 | 0 | 0 | 0 | 0.003 | 0 | 0.003 | 0.004 | 0 | 0 | 0 |
|  | Candidate_division_OP3 | 0.019 | 0 | 0 | 0 | 0 | 0 | 0.002 | 0.007 | 0.026 | 0 |
|  | Candidate_division_OP8 | 0.341 | 0.006 | 0.007 | 0.008 | 0.019 | 0.039 | 0.327 | 0.079 | 0.142 | 0.040 |
|  | Candidate_division_SR1 | 0.196 | 0.002 | 0 | 0 | 0 | 0 | 0 | 0.033 | 0 | 0.011 |
|  | Candidate_division_TM7 | 0.013 | 0.004 | 0 | 0.005 | 0 | 0 | 0 | 0 | 0 | 0 |
|  | Chlorobi | 0.171 | 0.054 | 0.056 | 0.179 | 0.043 | 0.036 | 0.114 | 0.007 | 0.019 | 0.017 |
|  | Chloroflexi | 6.292 | 0.239 | 0.689 | 0.886 | 0.286 | 0.929 | 2.098 | 0.361 | 0.699 | 0.350 |
|  | Cyanobacteria | 0.329 | 0.056 | 0.049 | 0.039 | 7.816 | 6.560 | 4.204 | 3.160 | 4.008 | 2.785 |
|  | Deferribacteres | 0.341 | 0 | 0 | 0 | 0.006 | 0.003 | 0.004 | 0.013 | 0.045 | 0.011 |
|  | Deinococcus-Thermus | 0.032 | 0.010 | 0 | 0.011 | 0 | 0.003 | 0 | 0 | 0.006 | 0.006 |
|  | Elusimicrobia | 0 | 0.027 | 0.014 | 0.008 | 0.360 | 0.093 | 0.093 | 0.007 | 0 | 0 |
|  | Fibrobacteres | 0 | 0.039 | 0.007 | 0.003 | 0 | 0 | 0 | 0 | 0 | 0 |
|  | Firmicutes | 8.069 | 18.009 | 20.769 | 22.485 | 43.155 | 54.223 | 43.288 | 74.103 | 68.383 | 63.468 |
|  | Fusobacteria | 0.006 | 0.035 | 0.014 | 0.008 | 0.068 | 0.023 | 0.002 | 0.079 | 0 | 0 |
|  | Gemmatimonadetes | 0.095 | 0.013 | 0.007 | 0.011 | 0 | 0 | 0.008 | 0.020 | 0.013 | 0.011 |
|  | Hyd24-12 | 0.525 | 0 | 0 | 0 | 0.006 | 0.010 | 0 | 0.013 | 0.013 | 0.006 |
|  | Lentisphaerae | 0.108 | 0.142 | 0.330 | 0.181 | 0.137 | 0.085 | 0.233 | 0.013 | 0 | 0.017 |
|  | Nitrospirae | 0.038 | 0 | 0 | 0 | 0.012 | 0 | 0.002 | 0.052 | 0.013 | 0.006 |
|  | Planctomycetes | 2.416 | 0.177 | 0.184 | 0.134 | 0.130 | 0.090 | 0.172 | 0.157 | 0.220 | 0.235 |
|  | Alphaproteobacteria | 11.155 | 1.016 | 0.410 | 0.273 | 4.141 | 2.488 | 1.581 | 1.036 | 1.800 | 0.951 |
|  | Betaproteobacteria | 1.322 | 0.296 | 0.177 | 0.134 | 0.019 | 0.013 | 0.018 | 0.426 | 0.227 | 0.155 |
|  | Deltaproteobacteria | 6.672 | 14.904 | 20.456 | 11.157 | 21.394 | 15.610 | 18.114 | 2.249 | 2.357 | 2.876 |
|  | Epsilonproteobacteria | 0.386 | 1.684 | 1.495 | 0.507 | 1.136 | 2.441 | 0.690 | 0.118 | 0.097 | 0.097 |
|  | Gammaproteobacteria | 12.332 | 17.234 | 12.061 | 8.675 | 0.168 | 0.159 | 0.191 | 1.541 | 1.891 | 0.991 |
|  | Other Proteobacteria | 1.549 | 0.296 | 0.563 | 0.305 | 0.453 | 0.818 | 1.138 | 0.072 | 0.110 | 0.052 |
|  | SHA-109 | 0.006 | 0 | 0 | 0 | 0 | 0 | 0 | 0 | 0 | 0 |
|  | Spirochaetae | 2.397 | 8.986 | 7.877 | 8.134 | 4.470 | 2.868 | 5.984 | 6.058 | 11.415 | 20.358 |
|  | Synergistetes | 0.076 | 0.472 | 0.417 | 0.302 | 0.317 | 0.412 | 0.513 | 0.052 | 0.058 | 0.023 |
|  | TM6 | 0.013 | 0 | 0 | 0.005 | 0.068 | 0.286 | 0.146 | 0 | 0.013 | 0 |
|  | Tenericutes | 0.841 | 0.566 | 0.254 | 0.368 | 0.596 | 0.365 | 0.438 | 0.538 | 0.544 | 0.183 |
|  | Thermotogae | 0 | 0 | 0 | 0 | 0 | 0 | 0 | 0.007 | 0.006 | 0 |
|  | Verrucomicrobia | 0.247 | 0.040 | 0.045 | 0.055 | 0 | 0.008 | 0.004 | 0.039 | 0.065 | 0.940 |
|  | WCHB1-60 | 0.006 | 0 | 0 | 0 | 0 | 0.005 | 0.002 | 0.007 | 0.013 | 0.011 |
|  | Unclassified^a^ | 14.260 | 4.907 | 4.246 | 6.625 | 2.223 | 1.608 | 2.490 | 1.364 | 1.858 | 1.501 |
|  |  |  |  |  |  |  |  |  |  |  |  |
| WWT | **Archaeal phyla/classes** | | | | | | | | | | |
|  | Methanomicrobia | 0 | 0 | 99.664 | 98.806 | 0 | 0 | 0 | 0 | 0 | 0 |
|  | Thaumarchaeota | 0 | 0 | 0.336 | 0.597 | 0 | 0 | 0 | 0 | 0 | 0 |
|  | Bathyarchaeota | 0 | 0 | 0 | 0.597 | 0 | 0 | 0 | 0 | 0 | 0 |
|  | **Bacterial phyla/classes** | | | | | | | | | | |
|  | Acidobacteria | 0.107 | 0.165 | 0.144 | 0.181 | 0.270 | 0.300 | 0.148 | 0.098 | 0.078 | 0.015 |
|  | Actinobacteria | 0.181 | 0.412 | 0.238 | 0.299 | 0.301 | 0.218 | 0.270 | 0.167 | 0.603 | 1.604 |
|  | Aquificae | 0 | 0 | 0 | 0 | 0 | 0 | 0.002 | 0 | 0 | 0 |
|  | Armatimonadetes | 0 | 0 | 0 | 0 | 0 | 0.011 | 0.019 | 0 | 0 | 0 |
|  | BD1-5 | 0.008 | 0.059 | 0.536 | 0.115 | 0.603 | 0.029 | 0.002 | 0.006 | 0.007 | 0 |
|  | Bacteroidetes | 43.485 | 17.761 | 33.054 | 39.128 | 33.590 | 26.415 | 33.460 | 5.729 | 4.742 | 1.680 |
|  | Caldiserica | 0.008 | 0.006 | 0.009 | 0.024 | 0 | 0.014 | 0.021 | 0 | 0 | 0 |
|  | Candidate_division_BRC1 | 0.049 | 0.012 | 0.008 | 0.030 | 0 | 0.018 | 0.021 | 0 | 0 | 0 |
|  | Candidate_division_OD1 | 0 | 0 | 0 | 0 | 0 | 0 | 0.002 | 0 | 0 | 0 |
|  | Candidate_division_OP3 | 0.008 | 0 | 0 | 0 | 0 | 0.004 | 0.014 | 0.002 | 0 | 0 |
|  | Candidate_division_OP9 | 0 | 0 | 0 | 0 | 0 | 0.007 | 0 | 0 | 0 | 0 |
|  | Candidate_division_SR1 | 0 | 0 | 0.028 | 0.012 | 0.021 | 0 | 0 | 0 | 0 | 0 |
|  | Candidate_division_TM7 | 0 | 0.006 | 0.003 | 0 | 0 | 0.004 | 0 | 0 | 0 | 0 |
|  | Chlamydiae | 0 | 0 | 0.001 | 0.006 | 0 | 0 | 0 | 0 | 0 | 0 |
|  | Chlorobi | 1.214 | 0.318 | 0.789 | 1.556 | 0.301 | 0.868 | 1.345 | 0.018 | 0.005 | 0.002 |
|  | Chloroflexi | 0.312 | 0.300 | 0.247 | 0.522 | 0.229 | 0.500 | 0.655 | 0.002 | 0.007 | 0.002 |
|  | Cyanobacteria | 0.304 | 15.184 | 0.030 | 0.100 | 0.042 | 13.084 | 4.629 | 0.207 | 0.243 | 0.091 |
|  | Deferribacteres | 0 | 0 | 0 | 0 | 0 | 0 | 0 | 0 | 0.005 | 0.002 |
|  | Deinococcus-Thermus | 0 | 0 | 0.004 | 0.006 | 0.021 | 0 | 0 | 0.002 | 0 | 0.002 |
|  | Elusimicrobia | 0.107 | 0.271 | 0.129 | 0.066 | 0.083 | 0.214 | 0.288 | 0.046 | 0.026 | 0.006 |
|  | Fibrobacteres | 0 | 0 | 0.015 | 0.012 | 0.021 | 0 | 0 | 0.002 | 0.005 | 0.002 |
|  | Firmicutes | 9.608 | 9.260 | 10.911 | 13.096 | 12.384 | 11.462 | 7.879 | 20.142 | 28.622 | 68.106 |
|  | Fusobacteria | 0 | 0.035 | 0.016 | 0.012 | 0.042 | 0.007 | 0.008 | 0.419 | 0.482 | 0.088 |
|  | Gemmatimonadetes | 0 | 0 | 0.001 | 0 | 0 | 0 | 0 | 0 | 0 | 0 |
|  | Lentisphaerae | 1.748 | 0.665 | 0.771 | 0.501 | 0.758 | 0.593 | 1.415 | 0.026 | 0.019 | 0.013 |
|  | Planctomycetes | 0.041 | 0.159 | 0.044 | 0.039 | 0.042 | 0.125 | 0.247 | 0 | 0 | 0.006 |
|  | Alphaproteobacteria | 0.139 | 0.482 | 0.279 | 0.383 | 0.281 | 0.357 | 0.369 | 0.789 | 0.411 | 0.095 |
|  | Betaproteobacteria | 0.788 | 1.565 | 1.271 | 0.661 | 1.932 | 1.065 | 0.527 | 6.483 | 5.777 | 2.420 |
|  | Deltaproteobacteria | 10.535 | 33.527 | 25.414 | 9.199 | 30.556 | 12.387 | 4.808 | 0.514 | 0.553 | 0.181 |
|  | Epsilonproteobacteria | 3.307 | 2.865 | 2.795 | 3.867 | 3.158 | 3.724 | 1.689 | 39.258 | 37.155 | 11.075 |
|  | Gammaproteobacteria | 0.673 | 2.077 | 1.141 | 0.947 | 2.016 | 0.829 | 0.396 | 25.192 | 20.510 | 14.443 |
|  | other Proteobacteria | 0.246 | 0.141 | 0.228 | 0.042 | 0.052 | 1.762 | 0.976 | 0.032 | 0.021 | 0.011 |
|  | SHA-109 | 0 | 0 | 0 | 0 | 0 | 0 | 0 | 0 | 0.002 | 0 |
|  | Spirochaetae | 14.990 | 6.554 | 11.841 | 19.077 | 5.330 | 13.846 | 28.463 | 0.054 | 0.026 | 0.015 |
|  | Synergistetes | 2.117 | 2.577 | 4.304 | 3.818 | 3.626 | 4.392 | 3.761 | 0.287 | 0.213 | 0.027 |
|  | TM6 | 0.016 | 0 | 0.005 | 0.003 | 0 | 0.007 | 0 | 0 | 0 | 0 |
|  | Tenericutes | 0.090 | 0.094 | 0.054 | 0.247 | 0.094 | 0.082 | 0.101 | 0.006 | 0.005 | 0 |
|  | Thermotogae | 0.131 | 0.018 | 0.049 | 0.178 | 0 | 0.025 | 0.492 | 0 | 0 | 0 |
|  | Verrucomicrobia | 1.173 | 0.471 | 0.177 | 0.247 | 0.208 | 0.590 | 0.857 | 0.150 | 0.116 | 0.032 |
|  | Unclassified^a^ | 8.615 | 5.018 | 5.465 | 5.625 | 4.042 | 7.059 | 7.137 | 0.369 | 0.366 | 0.082 |
|  |  |  |  |  |  |  |  |  |  |  |  |
| GL | **Bacterial phyla/classes** | T_0_ | Chara | | | Chlorella | | | Kelp | | |
|  |  |  | T_4_ | T_8_ | T_13_ | T_4_ | T_8_ | T_13_ | T_4_ | T_8_ | T_13_ |
|  | Acidobacteria | 0.146 | 0.003 | 0 | 0 | 0.016 | 0.009 | 0.046 | 0 | 0.881 | 0.338 |
|  | Actinobacteria | 1.696 | 0.074 | 0.014 | 0.063 | 0.275 | 0.176 | 0.127 | 0.758 | 1.712 | 0.782 |
|  | Aquificae | 0 | 0.003 | 0 | 0.006 | 0 | 0.005 | 0 | 0 | 0 | 0.011 |
|  | BD1-5 | 0 | 0 | 0 | 0 | 0 | 0 | 0 | 0 | 0 | 0.011 |
|  | Bacteroidetes | 0.804 | 7.304 | 12.498 | 13.583 | 7.731 | 10.226 | 10.693 | 6.061 | 3.979 | 1.093 |
|  | Candidate_division_BRC1 | 0 | 0 | 0 | 0.006 | 0 | 0 | 0 | 0 | 0.176 | 0.039 |
|  | Candidate_division_OP10 | 0.041 | 0 | 0 | 0.046 | 0 | 0.009 | 0 | 0 | 0.050 | 0.078 |
|  | Candidate_division_OP8 | 0 | 0 | 0 | 0 | 0 | 0 | 0 | 0 | 0 | 0.006 |
|  | Candidate_division_SR1 | 0.024 | 0 | 0 | 0 | 0 | 0 | 0 | 0 | 0 | 0 |
|  | Candidate_division_TM6 | 0.024 | 0 | 0 | 0.006 | 0 | 0 | 0 | 0 | 0.025 | 0.044 |
|  | Candidate_division_TM7 | 0 | 0.003 | 0 | 0 | 0 | 0 | 0 | 0 | 0 | 0 |
|  | Candidate_division_WS3 | 0.041 | 0 | 0 | 0 | 0 | 0.005 | 0.004 | 0 | 0.227 | 0.100 |
|  | Chlamydiae | 0.032 | 0 | 0 | 0 | 0 | 0.005 | 0.004 | 0 | 0.025 | 0.033 |
|  | Chlorobi | 0.081 | 0 | 0 | 0 | 0 | 0.005 | 0.039 | 0 | 0.025 | 0.044 |
|  | Chloroflexi | 0.260 | 0.012 | 0.043 | 0.006 | 0.024 | 0.036 | 0.039 | 0 | 0.680 | 0.233 |
|  | Cyanobacteria | 5.008 | 0.113 | 0.187 | 0.108 | 0 | 0 | 2.260 | 19.700 | 35.030 | 22.910 |
|  | Deferribacteres | 0.008 | 0 | 0 | 0 | 0 | 0 | 0 | 0 | 0.050 | 0.055 |
|  | Deinococcus-Thermus | 0.057 | 0.003 | 0 | 0 | 0 | 0 | 0 | 0 | 0 | 0 |
|  | Fibrobacteres | 0.008 | 0 | 0 | 0 | 0 | 0 | 0 | 0 | 0 | 0 |
|  | Firmicutes | 0.390 | 14.789 | 20.778 | 19.015 | 21.834 | 11.871 | 9.781 | 25.758 | 20.927 | 60.568 |
|  | Fusobacteria | 0.008 | 0 | 0 | 0 | 0.016 | 0 | 0.028 | 0 | 0 | 0.006 |
|  | Gemmatimonadetes | 0.073 | 0 | 0 | 0 | 0 | 0.005 | 0.004 | 0 | 0.025 | 0.128 |
|  | Lentisphaerae | 0.008 | 0 | 0 | 0 | 0.008 | 0 | 0 | 0 | 0.076 | 0.033 |
|  | NPL-UPA2 | 0 | 0 | 0 | 0 | 0 | 0 | 0 | 0 | 0 | 0.006 |
|  | Nitrospirae | 0.008 | 0 | 0 | 0 | 0.008 | 0 | 0 | 0 | 0 | 0.055 |
|  | OPS8 | 0.008 | 0 | 0 | 0 | 0 | 0 | 0 | 0 | 0 | 0 |
|  | Planctomycetes | 0.528 | 0.027 | 0.014 | 0.023 | 0.146 | 0.072 | 0.063 | 3.788 | 3.274 | 2.008 |
|  | Alphaproteobacteria | 1.384 | 0.113 | 0.173 | 0.119 | 0.307 | 0.135 | 0.137 | 2.273 | 4.533 | 2.291 |
|  | Betaproteobacteria | 1.230 | 0.053 | 0.029 | 0.040 | 0.202 | 0.068 | 0.115 | 2.273 | 5.011 | 2.779 |
|  | Deltaproteobacteria | 0.436 | 6.481 | 6.911 | 8.458 | 14.734 | 18.956 | 22.254 | 16.667 | 2.367 | 1.032 |
|  | Epsilonproteobacteria | 0.094 | 0.003 | 0.029 | 0.011 | 0 | 0 | 0.011 | 4.545 | 0.252 | 0.006 |
|  | Gammaproteobacteria | 91.823 | 71.014 | 59.294 | 58.472 | 54.569 | 56.792 | 53.979 | 17.424 | 19.214 | 4.627 |
|  | Other Proteobacteria | 0 | 0.003 | 0 | 0 | 0 | 0 | 0 | 0 | 0.025 | 0 |
|  | Spirochaetes | 0.106 | 0 | 0.029 | 0.023 | 0.081 | 1.582 | 2.007 | 0 | 0.176 | 0.011 |
|  | Synergistetes | 0.016 | 0 | 0 | 0 | 0 | 0 | 0 | 0 | 0.076 | 0.006 |
|  | Thermodesulfobacteria | 0.008 | 0 | 0 | 0 | 0 | 0 | 0 | 0 | 0 | 0 |
|  | Verrucomicrobia | 0.390 | 0.003 | 0 | 0.017 | 0.040 | 0.045 | 0.141 | 0.758 | 1.184 | 0.671 |
|  | WCHB1-60 | 0.016 | 0 | 0 | 0 | 0.008 | 0 | 0 | 0 | 0 | 0 |
|  | Unclassified^a^ | 0 | 0.012 | 0 | 0.017 | 0 | 0 | 0 | 0 | 0 | 0 |
|  |  |  |  |  |  |  |  |  |  |  |  |

a: The unclassified bin include those sequences that were not classified into a phylum with ≥80% confidence using the mothur software classify.seqs command. Note that the percentage of unclassified sequences decreased with enrichment mainly due to the enrichment in the percentages of a few phyla that are considered significant for the algal degradation process. The analysis presented in the study was mainly focused on the sequenced that were classified with confidence.

**Supplementary figures:**

**Figure S1**. Chlorophyll-a levels and oxygen concentration along depth in Grand Lake sites used as inoculum source in this study. Chlorophyll A concentration is shown as 🞎 on the primary X-axis, while average dissolved O2 is shown as ◇ on the secondary X-axis. Boxed region depicts the depth at which the samples were obtained for this study.


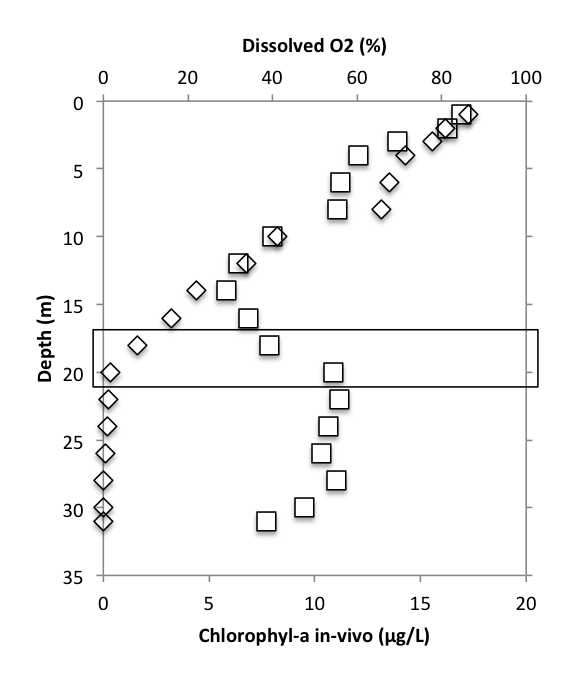


**Figure S2.** Change in sulfate concentration with enrichment time in ZDT (A), WWT (B), and GL (C) enrichments on Chara (△), Chlorella (□), and Kelp (◇).


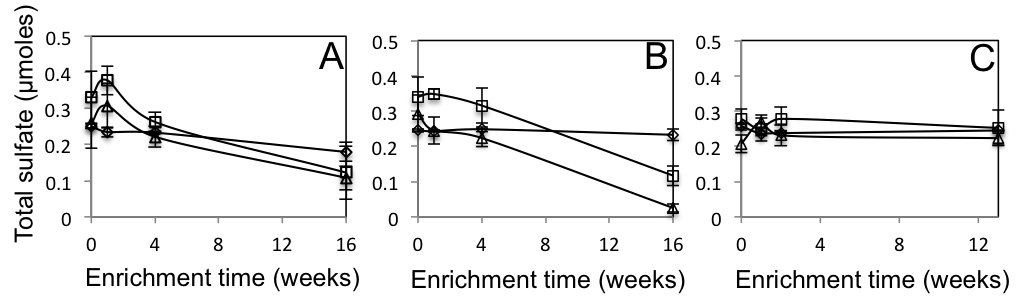

Supplement: Supplemental Information 1 — Includes two supplementary tables and two supplementary figures Table S1 Table S2 Figure S1 Figure S2 [file peerj-05-2803-s001.docx]
